# Autism and Probable Prerequisites: Severe and Scheduled Prenatal Stresses at Spotlight

**Published:** 2018-09

**Authors:** Seyyed Mohammad MOUSAVI, Elahe KAMALI, Fatemeh FATAHI, Hadi BABAIE, Mansoor SALEHI

**Affiliations:** 1. Cellular and Molecular Research Center, Shahrekord University of Medical Sciences, Shahrekord, Iran; 2. Genetic and Identification Lab, Legal Medicine Center, Isfahan, Iran; 3. Division of Genetics, Dept. of Biology, Faculty of Sciences, University of Isfahan, Isfahan, Iran; 4. Dept. of Genetics and Molecular Biology, School of Medicine, Isfahan University of Medical Sciences, Isfahan, Iran; 5. Genetic and Identification Lab, Legal Medicine Center, Rasht, Iran; 6. Medical Genetics Center of Genome, Genome Building, Isfahan, Iran

**Keywords:** Autism, Prenatal stress, Epigenetic

## Abstract

**Background:**

Due to the importance of prenatal maternal stress as environmental factor on autism, the influence of prenatal maternal psychological agitations was assessed in relation with the risk of autism.

**Methods:**

In this case-control study, some mothers of autistic children in Isfahan, central Iran, in 2014, were retrospectively compared with control mothers in terms of quantity, quality, and schedule of exposure to 45 stressful events in a 15-month period. In addition, dividing the stressors into two groups of genome-dependent/independent events, their prevalence was separately scrutinized and compared among patient and control families.

**Results:**

Although the child’s risk of autism increases significantly with the increase of maternal stress during months 4–7 of pregnancy, the increased stress during months 2–3 of pregnancy can lead to a significant increase in the severity of autism affliction as well as a slight but significant increase in the possibility of LFA in afflicted children (*P*<0.05). The overall prevalence of genome-dependent stressful events among two patient and control groups was significantly higher than that of genome-independent events (*P*=0.000), but genome-dependent events led to more stress inpatient families.

**Conclusion:**

Although the present study consistent with recent findings in the fields of epigenetics and gene-environment interactions can confirm the role of severe and scheduled prenatal stresses in causing autism, it does not deny the necessity of a perspective and wider study in Isfahan and Iran.

## Introduction

Impaired social interaction, speech disorders, stereotyped behaviors and irregularity in the pattern of interest are main characteristics that in a male child under 3 are highly indicative of autism (1).

Despite the estimates of previous twin (2) and association (3–5) studies, recently the involvement of genetic factors was estimated in causing autism less than previous studies (37% responsibility for autism and 38% for ASD) and the role of common environmental factors before and after the birth of twins in causing autism much more important than that in previous estimates and even more than genetic factors (55% liability for autism and 58% for ASD) (6).

Prenatal maternal psychological stress is one of the environmental clues becoming progressively and absolutely associated with autism almost a decade after the first report on it (7). Often mothers of autistic children were more exposed to Holmes-Rahe ranked stressful events during their pregnancy, predominantly during wk 21–32 with a peak during wk 25–28 compared to control mothers (8).

In addition, the prevalence of autism was increased in people exposed more severely, to hurricanes and tropical storms (26.59 in 10000) (during prenatal months 5–6 (wk 19–27) and 8–9 (wk 32–36)) (9, 10); and to stressful events (10) in Louisiana than that in people less exposed to this event (3.72 in 10000) (9).

On the other hand, to reject this relationship, two large population studies did not show any significant relationship between increased risk of autism and pregnant mothers’ exposure, to bereavement (11), and to the low/high-incidence stressful events (12).

In the present study, we scrutinized the hypothesis of the increased risk of autism in the event of prenatal maternal psychological agitations. In addition, we measured and compared the effects of prevalence, severity, nature and timing of exposure to 45 stressful events between 74 mothers of autistic children and 69 control mothers.

## Materials and Methods

### Participants

Until 2014, the start of our study, 202 families having at least one autistic child were identified via DSM-IV-TR and organized at the Department of Rehabilitation of State Welfare Organization of Isfahan City, central Iran.

Using the Cochran’s sample size formula with the confidence level of 0.05 and considering the population size of families with autistic children in Isfahan province (N=202), the sample size of 86 was calculated; thus, two groups of case and control, each with 86 families entered this case-control study. From these families, 12 families in the case group and 17 families in the control group were on exclusion criteria and were excluded from the study. Finally, the sample size was decreased to 74 in the case group and to 69 in the control group.

All the both group’s parents filled out the consent form to participate in this study according to the protocol of the Ethical Review Board of the Behavioral Sciences Research Center of Isfahan University of Medical Sciences.

Autism was diagnosed based on the 4^th^ revised edition of Diagnostic and Statistical Manual (DSM-IV-TR) and the 10^th^ International Classification of Diseases (ICD-10).

As to the control group, the children (the most recent child in the family and matched to the affected children in terms of age and socioeconomic status) had no records of apparent, verbal and neurodevelopmental disorders. However, up to 8 families were excluded from the study due to reporting a history of psychiatric disorders in their children.

### Procedures

Data were collected from both groups using a questionnaire containing 45 stressful events regarding a period containing 6 months before pregnancy to the moment of birth. Both parents of autistic children were separately questioned to clarify this 15-month period of time. Then weighted prevalence and severity of these stressful events were compared between the two affected and control groups.

The questionnaire applied was designed based on the ranking of stressful life events (13) (adapted Holmes-Rahe stressful events (14) to Iran’s cultural-social conditions). However, some cases stated such as menopause were removed from the study due to the unlikelihood of its occurrence in studied families, and some cases such as living in father-in-law’s house not existing in the questionnaire were added.

To adjust the severity of stressful events and compare the total numerical amount of stress received by each mother between two groups, the total weighted life event score for each stressful event based on the ranking was separately calculated and compared between two groups (13). Rate of forgetting the stressful events of Holmes-Rahe questionnaire (more than 90% reflected in the questionnaire of Malek et al.) is very low (15). In addition to all stresses received by the mother, stressful events were divided into two groups of genome-dependent events (events resulting from genome-environment interactions) and genome-independent events (purely environmental). The level of prenatal stress affecting the maternal genes depends on the nature of the stressful event. In fact, the incidence of some stressful events such as divorce, physical illnesses or family problems (dependent stressors) *might be* directly or indirectly attributed to maternal genotype, but some other stressful events such as storm or war can never be attributed to maternal genotype and occur quite independently (independent stressors). Accordingly, 30 events (62.5%) and 15 events (37.5%) of 45 events examined in this study were identified as genome-dependent & independent stressors, respectively. The prevalence and severity of both of these categories were separately analyzed between patient and control groups.

Finally, the collected data were analyzed using SPSS (ver.17, Chicago, IL, USA) and independent sample t-test, Chi-square (χ^2^), Fisher exact test and Logistic Regression analysis were also used.

## Results

Contrary to the most studies, gender ratio of afflicted children was much larger than expected, the ratio of 2.7 boys (72.6%) to 1 girl (27.4%). The mean education level of mothers (*P*>0.05), unwanted pregnancies (*P*>0.05) and aggressive behaviors (*P*<0.05) was higher in patient group (Table 1).

**Table 1:** Frequency distribution of demographic characteristics in two study groups

***Variables***		***Control (n=69)(%)***	***Case(n=74) (%)***	***P-value***
Count offspring	1	20(28.8)	26(35.1)	0.582
	2 or 3	46(69.7)	45(60.8)	
	≥4	3(4.5)	3(4.1)	
Age(yr)	Maternal age at delivery of child	31.56±0.68	25.94±0.53	<0.001
Gender of child	Boy (autistic)	40(58)	53(72.6)	0.338
	Girl (autistic)	29(42)	20(27.4)	
Mother’s education level	<diploma	18(26.5)	17(23.9)	0.205
	Diploma	35(51.5)	30(42.3)	
	F diploma	8(11.8)	7(9.9)	
	B.S	7(10.3)	17(23.9)	
Aggression	Yes	9(13.4)	39(57.4)	<0.001
	No	58(86.6)	29(42.6)	
Pregnancy	Yes	55(79.7)	48(66.7)	0.060
	No	14(20.3)	24(33.3	
Autism Severity	LFA	-	41(56.9)	-
	HFA	-	31(43.1)	

**Fig. 1:**
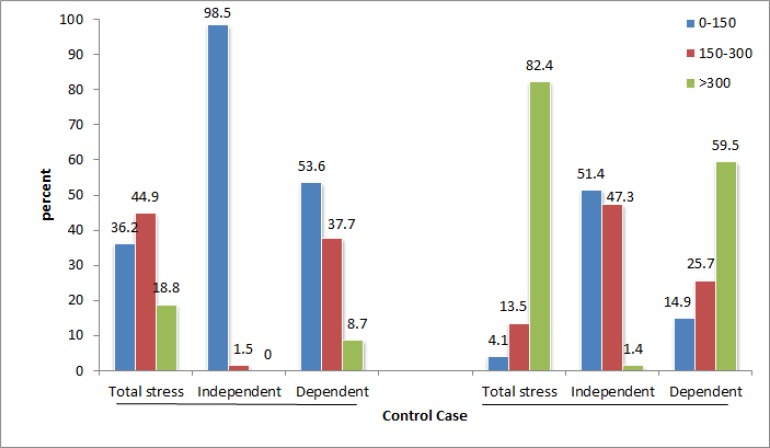
The two-way frequency bar graph of groups sensitive to disorders caused by stress in the two study groups

In average, each affected family encountered 9.71 (21.5%) stressful events in this 15-month period, while this rate in control group was significantly lower and about 4.63 (10.28%) (*P*<0.05). “Unhappy marriage” and “failure to achieve desired goals of life” are very high-incidence events in afflicted group (Table 2). The overall mean weighted scores for stress inpatient group and control group were 516.71±25.44 and 183.38±12.8, respectively and shows a very significant difference (*P*<0.05) (Table 3). Moreover, the prevalence and severity of genome-dependent stressors in both groups are higher than the genome-independent stressors (Table 3 and Fig. 1).

**Table 2:** Determination and comparison of mean high-incidence stressful events in patient group compared to control group

***Factors***	***Control(n=69) Mean±SD***	***Case(n=74) Mean±SD***	***P-value***
Unhappy marriage	0.52±3.06	6.61±8.87	0.000^*^
Occurrence of an unexpected event	0±0	1.15±4.31	0.028^*^
Failure to achieve desired goals of life	1.67±5.02	6.25±8.07	0.000^*^
Health impairment of a family member	0.66±3.14	2.90±6.04	0.007^*^
Major change in financial status	2.34±5.43	4.97±7.01	0.014^*^
Mother’s serious illness or injury	0.60±2.85	1.20±3.93	0.000^*^
Frequent arguments with spouse	1.20±3.93	4.68±6.60	0.000^*^
Undesirable workplace conditions	0.19±1.59	3.93±6.09	0.000^*^
Lack of security	0.18±1.49	1.67±4.27	0.007^*^
Having to change one’s lifestyle	0.52±2.45	2.42±4.84	0.004^*^
Major change in the sleeping habits	1.58±3.86	3.53±5.14	0.012^*^
Sexual problems	1.04±3.13	2.64±4.53	0.016^*^
Reconciliation with spouse	0.23±1.32	1.59±3.18	0.001^*^
Unintentional miscarriage	0.47±1.91	1.42±3.11	0.030^*^
Marriage	0.27±1.60	1.67±3.64	0.004^*^
Nowruz	1.64±2.68	3.46±2.96	0.000^*^
Obsession	1.76±4.91	4.32±6.92	0.012^*^
Living in father-in-law’s house	2.18±5.35	5.91±7.42	0.001^*^
Stress due to various reasons	0±0	7.58±5.12	0.000^*^
Pregnancy without mother’s notice	0±0	1.12±3.81	0.016^*^

*P*<0.05, therefore there is a significant difference between the two groups

**Table 3:** Compare score of stress between two groups study

***Factors***	***Control (n=69)***	***Case(n=74)***	***P-value***
Total Stress	183.38±12.80	516.71±25.44	<0.001
Independent	36.00±5.28	141.33±8.97	<0.001
Dependent	141.41±12.22	375.38±23.72	<0.001

Therefore, according to the triple classification of people encountering stress suggested by Holmes-Rahe (14), “moderate susceptibility to stress-related disorders” had the highest frequency in control group and both of them were classified as “low susceptibility to stress-related disorders” in terms of genome-dependent and independent stressful events (Diagram 1). However, “high susceptibility to stress-related disorders” had the highest frequency in patient group in terms of both the overall received stress and genome-dependent stressors and these mothers were included in the range of “moderate susceptibility to stress-related disorders” only in terms of genome-independent stressors. There is significant relationship using Chi-square test among the types of sensitivity to stress-related disorders between the two groups (*P*<0.05) (Fig. 1).

The frequency distribution of stress incidence from 6 months before pregnancy to the moment of birth (month 9) is presented in Table 4. The pairwise comparison of groups in all months of pregnancy showed that there was a notable and significant difference between the two groups in months 4, 5, 6 and 7 (*P*<0.01), so that during these months, the stress incidence in the group having autistic child is much higher than that in control group (Table 4).

**Table 4:** Frequency distribution of stress incidence and severity from 6 months before pregnancy to month 9 in the two groups

***Month***	***Case***		***P-value^a^***	***Control (%)***	***P-value^b^***
	***LFA (%)***	***HFA(%)***			
Month -6	0(0)	1(100)	0.574	0(0)	0.734
Month -5	0(0)	2(100)	0.325	2(2.9)	0.663
Month -4	2(50)	2(50)	0.574	2(1.9)	0.374
Month -3	4(66.7)	2(33.3)	0.206	3(5.7)	0.283
Month -2	4(50)	4(50)	0.467	4(6.8)	0.219
Month -1	4(50)	4(50)	0.467	4(5.8)	0.219
Month 1	16(51.6)	15(48.4)	0.118	21(30.4)	0.106
Month 2	18(56.2)	14(43.8)	0.049^*^	25(36.2)	0.247
Month 3	19(52.8)	17(47.2)	0.047^*^	27(39.1)	0.164
Month 4	17(47.2)	19(52.8)	0.272	18(26.1)	0.004^*^
Month 5	16(44.4)	20(55.6)	0.468	18(26.1)	0.004^*^
Month 6	17(39.5)	26(60.5)	0.318	19(27.5)	<0.001^*^
Month 7	14(36.8)	24(63.2)	0.182	25(36.2)	0.049^*^
Month 8	13(38.2)	21(61.8)	0.302	25(36.2)	0.156
Month 9	12(40)	18(60)	0.441	31(44.9)	0.359

a: The difference between LFA (Low Functioning Autism) and HFA (High Functioning Autism) in case group,

b: The difference between two groups (case and control),

*: *P*<0.05 denote significant difference between two groups

Mothers having been exposed to more stress in months 2 and 3 of pregnancy are more likely to have LFA (Low Functioning Autism) children.

This difference was considered significant using Fisher exact test (*P*<0.01). In other words, the exposure of mothers to stress in months 2 and 3 of pregnancy may increase the probability of newborns with more severe autism, if their exposure is continued and intensified in the following months (especially month 6 of pregnancy).

## Discussion

This retrospective study aimed to analyze the probable relationship between the increased quantity and intensity of stresses received by the mother during pregnancy and the increased risk of autism. This relationship was strongly perceived in this study, in both genders. A significant increase of exposure to stressful events during months 4–7 (wk 14–32) of pregnancy was observed only in autistic children. Therefore, possibly we can suggest that the stress incidence in months 4–7 of pregnancy can increase the risk of autism (Table 4).

However, this increased exposure was reported during months 5.5–7 (wk 21–32) of pregnancy (8). In addition, months 5–6 (wk 19–27) and 8–9 (wk 32–36) were introduced as the most critical period of pregnancy to evolve autism, so that the exposure of mothers to severe stress during this period increases the risk of autism in the child 3.83 times more than other periods of pregnancy (9).The patient group received the peak stress in month 6 (wk 23–27) of pregnancy, which is highly consistent with the previous study (8) (wk 25–28). However, Beversdorf et al. have suggested the period of wk 21–32 of pregnancy in their study (8). Investigation of factors affecting the stress severity in patient group showed that the stress severity decreases approximately with increasing levels of the mother’s education. Infants experiencing higher prenatal stress have represented more aggressive behaviors; but, none of these factors have affected the stress severity.

Although the child’s risk of autism increases significantly with the increase of maternal stress during months 4–7 (wk 14–32) of pregnancy, the results of our study show that the increased stress during months 2–3 (wk 5–13) of pregnancy leads to a significant increase in the severity of autism affliction as well as a slight but significant increase in the possibility of LFA in afflicted children (*P*<0.05).

Dividing the stressful events into two groups of genome-dependent and genome-independent events (purely environmental), the severity and scheduling of each group were separately scrutinized. The key question is how much is the potential of genome-dependent and genome-independent events to horse the correlation of prenatal stress with post developmental problems? For example, to separate purely genetic and purely environmental effects of prenatal stress, a cross-fostering study in rats and identifying ADHD in children was found that the relationship between prenatal stress and this behavioral disorder mostly results from genetic factors inheritance (16). In the present study, using the logistic regression analysis (Table 5) showed that both genome-dependent and independent stressors have significantly affected the stress among which the effect of genome-dependent stressors (β=3.76) had been more than that of genome-independent stressors (β=1.86), indicating the results of another study (16).

**Table 5:** Logistic regression analysis of factors affecting autism

***Factors***	***β***	***OR (95% CI)***	***P value***
genome-dependent stressors	3.76	42.86(4.3–421.65)	<0.001
genome-independent stressors	1.86	6.44(2.48–16.75)	0.001
Sex of child	2.60	0.074(0.01–0.40)	0.003
Maternal age at delivery child	0.26	0.76(0.66–0.88)	0.001

Furthermore, factors such as children’s gender (β=2.60) and mothers’ age (β=0.26) influence the occurrence of autism (*P*<0.05); in other words, the probability of girls becoming autistic is more than boys. On the other hand, young mothers giving birth are more probable to have autistic children (Table 5). In fact, these results show the higher potential of genome-dependent stressful events in accepting the responsibility of the association between prenatal stress and post devastating consequences in children.

A significant portion of life stresses in the society is unavoidable. However, the invasion of stressful events by a pregnant woman can outbreak as a variety of physical, behavioral and psychological disorders in her child; disorders such as asthma (17), cancer (18), drug abuse (19), schizophrenia (20), epilepsy (21), depression (22), abortion (23), learning deficits (24), mood disorders (25), low birth weight or high birth weight(26) and even the length of chromosomal telomeres in the middle age (27), etc. In fact, the important question is which factor (or factors) in a person having experienced prenatal stress or a kind of mismatch between embryonic conditions and postnatal conditions make him sensitive to a certain type of disease, not other types of it. Possible factors affecting the divergence of long-term effects of prenatal stress include: Differences genetic and epigenetic structures, difference in scheduling of exposure to stressful events, difference in types of these events, difference in duration of exposure to stress, difference in scheduling the identification of the effects of postnatal stressful events, sex moderating effects (sex differences in coping with stressful events) as well as difference in transgenerational accumulated epigenetic effects. Two factors of difference in scheduling the exposure to stress (28) and the Treasury of Accumulated Transgenerational Epigentic Effects (TATEE) are briefly described in the following.

### Difference in scheduling the exposure to stress

In a recent prospective study (29), the effects of high pregnancy anxiety (in wk 19, 25 and 31 of pregnancy) have been reported on the brains of 6–9-yr-old children. Physiological reflection of maternal stress can affect the fetus in a number of ways among which maternal and fetal HPA axes, as well as the placenta, are the most important candidate mechanisms (28, 30–32). However, studies show the human placenta has the most permeability towards corticosteroids near the middle of pregnancy (between wk 19–26 of pregnancy) and at the end of neurogenesis and on the onset of synaptogenesis (28).Autism is most likely to be developed during this critical and vulnerable period if large amounts of corticosteroids enter due to severe exposure of the mother to stressful events (9). This period is largely consistent with the period considered in our study (wk 14–32).

### Treasury of Accumulated Transgenerational Epigenetic Effects (TATEE):

High potential of epigenome in explaining patterns of health and disease is reported (33). As briefly as possible, TATEE (i.e. gathering and adding the biological effects of the exposure of several generations of organisms to different environmental factors affecting the epigenetic structures of the organism) (34) can certainly be a rich, important and emerging source of biological-historical information (35, 36). In fact, biological reflection of past environment can be transmitted to next generations through mechanisms such as transgenerational epigenetic effects (37) and time lapse can continuously add to this treasury that could predispose the organism to a range of illnesses.

## Conclusion

Environmental and neuropsychiatric condition of pregnant mothers can have profound and unpredictable effects on the fetus’s health. However, what was more elucidated here is that the severe and scheduled prenatal stress can predispose to autism, probably in a special condition.

## Ethical considerations

Ethical issues (Including plagiarism, informed consent, misconduct, data fabrication and/or falsification, double publication and/or submission, redundancy, etc.) have been completely observed by the authors.
